# Optimizing the Extraction Efficiency of Flaxseed Gum Using a Response Surface Methodology Approach

**DOI:** 10.1155/2024/5135565

**Published:** 2024-06-12

**Authors:** Abdessamad Ettouil, Asmaa Oubihi, Hamada Imtara, Khadija Atfaoui, Ramzi A. Mothana, Omar M. Noman, Mahmoud Tarayrah, Mohammed Ouhssine

**Affiliations:** ^1^Natural Resources and Sustainable Development Laboratory, Department of Biology, Faculty of Sciences, Ibn Tofail University, Bp: 133, Kenitra, Morocco; ^2^Faculty of Medicine, Arab American University Palestine, Jenin 44862, State of Palestine; ^3^Department of Pharmacognosy, College of Pharmacy, King Saud University, Riyadh 11451, Saudi Arabia; ^4^Groupe Hospitalier Cochin-Port Royal, Faculty of Medicine, Institut Cochin, Paris University, CNRS, IN-SERM, Paris 75000, France

## Abstract

The extraction of gum from natural raw materials is of increasing importance in various industries, including food, pharmaceuticals, and cosmetics, particularly due to their emulsifying properties and potential applications as stabilizers and thickeners. This study presents an insight on the influence of changing parameters like reagents and operating condition on yield and some properties of the flax (*Linum usitatissimum* L.) seed gum. The extraction conditions were meticulously examined using a full factorial design, highlighting the significant impact of pretreatment, seed preparation, and solvent selection on the extraction yield. A response surface methodology (RSM) was then applied to optimize the water/benzoic acid ratio of the pretreatment step, the ethyl alcohol/water ratio, and the medium pH of the extraction method, resulting in a maximum yield of 14.47%. Furthermore, detailed analyses of the chemical and emulsifying properties of the gum were conducted showing emulsifying capacities over 94%, offering promising application prospects, particularly in the food industry.

## 1. Introduction

Hydrocolloids extracted from plant sources, especially polysaccharide gums, have become indispensable ingredients in the food, pharmaceutical, and cosmetic industries. Their versatility as thickeners, stabilizers, gelling agents, and emulsifiers in food products, their role in the manufacture of controlled-release drugs, and their use in cosmetic products make them valuable components. Their appeal lies in their natural origin, availability, sustainability, and their ability to meet the growing consumer demand for natural and healthy ingredients [1].

Among the potential sources of these valuable gums, flax or linseed, from the botanical name *Linum usitatissimum* L. (Linnaeus, C., 1857), has attracted particular interest due to their abundance and rich polysaccharide composition. Flaxseeds, already widely recognized for their nutritional benefits because of their omega-3 fatty acids and dietary fiber content, offer a unique opportunity for the full utilization of plant-based raw materials [2]. Their polysaccharide content holds considerable potential for the production of high-quality polysaccharide gums. This abundant and economically viable source contributes to addressing the increasing demand for high-quality products [3].

Beyond its physical attributes which can be used as a thickening and emulsifying agent in food and other industries like pharmaceutic and cosmetic, flaxseed gum (FG) also known as flaxseed or linseed mucilage offers several health benefits [2]. It can be utilized as a dietary fiber source in human nutrition or act as a prebiotic, supporting the healthy growth of gut flora. In fermented dairy products, FG promotes the growth of lactic acid bacteria while positively influencing the product's texture additionally to the antimicrobial properties against pathogenic bacteria and fungi [4]. Moreover, in vivo studies have demonstrated the antiulcer activity of FG, reducing the number and length of gastric ulcers induced by ethanol in rats [5]. Chemical compounds derived from flaxseed exhibit antitumor properties due to their antioxidant activity, preventing the oxidation of proteins, lipids, or DNA, which are potential causes of cancer [6].

In Morocco, the flaxseeds are generally used as ingredients for herbal teas or served as gourmet dishes. Lahsissene et al. mentioned in the catalogue of medicinal plants used in the region Zaër (Western Morocco) that the species *L. usitatissimum* is highly recommended against urinary tract infections in the Zaër region [7]. For example, it is used at night in a mixture with other herbal teas, after decoction in water, as laxative and to treat constipation and urolithiasis [8, 9]. In a recent survey of medicinal plants used to treat hypercholesterolemia in Casablanca, it was found that the majority of herbalists frequently prescribe *L*. *usitatissimum* seeds, followed by *Coriandrum sativum* L., due to their higher use value. In addition, *L. usitatissimum* is reported to be one of the most marketed medicinal plants for its hypocholesterolemic properties, due to its richness in metabolites derived from the fatty acid, sterol, and lignan families [10]. The chosen extraction method is crucial to target the appropriate substances [4]. According to the herbalists, a convergence was noticed in the rate of methods used to prepare the plant extract, from infusion in 33% of cases to decoction or as powder in 30.3% of cases each. In agreement, the majority of surveys have reported the wide use of infusion and decoction for traditional drug preparation [11]. In popular pharmacopoeia, it is commonly prescribed as a laxative, emollient, and cough suppressant. Flaxseed is used to treat diabetes, heart disease, hypertension, and gastrointestinal and kidney disorders. A study on the effect of FG consumption in fasting subjects showed a lowering of total cholesterol (TC) and LDL-cholesterol, with an increase in fecal fat excretion [12].

Flaxseed carbohydrates are primarily concentrated in the hull, with a minimal 1–2% being digestible carbohydrate, mainly in the form of soluble sugar. The majority of flaxseed carbohydrates are indigestible, comprising both soluble and insoluble fiber. FG located in the outermost layer of the hull accounts for approximately a quarter of the total carbohydrate in flaxseed, making up about 7–10% of the total seed composition. The insoluble part of flaxseed carbohydrate is composed mainly of nonstarch polysaccharides, including cellulose and lignan [13, 14]. Structural studies reveal that FG consists of heterogeneous polysaccharides with a stiff random coil structure [15]. This mucilage is a complex mixture of polysaccharides, encompassing L-galactose, D-xylose, L-arabinose, L-rhamnose, and D-galacturonic acid, with a trace of D-glucose. Further division of FG reveals a neutral fraction (83%) and an acidic fraction (17%). The neutral fraction includes arabinoxylans (56%) and galactoglucans (44%), while the acidic fraction contains pectin-like molecules composed of L-rhamnose, D-galactose, L-fucose, D-xylose, L-arabinose, and D-glucose [16, 17]. Reportedly, the extraction conditions of FG significantly affect its composition, particularly its monosaccharide and protein contents.At high-temperature extraction range (70–90°C) an increase in the yield and the acidic polysaccharide is observed associated with the inactivation of microorganisms and enzymes, protein denaturation, and a decrease in the production of other compounds [18].

Nowadays, the extraction of FG presents a multifaceted challenge, requiring a balance between yield, purity, and cost-effectiveness. The current state of the art in FG extraction techniques involves a spectrum of methodologies aimed at optimizing yield and quality [19]. Conventional methods encompass solvent-based approaches, temperature variations, and pH adjustments, while more recent innovations explore enzymatic treatments or microwave-assisted extraction. Some studies focus on specific aspects like chemical composition, while others strive for environmentally friendly processes. Despite the diversity of approach, common threads emerge among these techniques. They all seek to disrupt the structural integrity of the flaxseed matrix to release the gum components effectively. Safdar et al. reported that the traditional hot water extraction technique (HWE), which is easy to control and requires no special equipment or conditions, achieved a higher yield (8.92%) at around 80°C compared to other methods like ultrasound-assisted (UAE) and alkaline-acidic extraction (AAE). However, UAE is more conducive to FG purity [20]. Nevertheless, these methods require a lot of solvent and other reagents despite their efficiency in terms of yield and feasibility on a small scale. The need for a cost-effective and efficient extraction method is crucial for the utilization of FG.

Statistical approaches are recommended to address the complexities of optimizing extraction conditions. The response surface methodology (RSM) designs, specifically central composite design (CDD), have proven to be invaluable tools in scientific research in various fields, from chemistry to engineering when a more comprehensive exploration of the experimental space is required [21]. However, the Box–Behnken design provides a balanced compromise between accuracy and practicality, particularly suitable for exploring complex relationships among continuous variables with a structured approach to identify optimal conditions while minimizing experimental trials, thus saving time and resources [22]. Its three-level design provides precise insights into process responses based on multiple factors [23].

This study aims to expand our understanding of the impact of changing pretreatment and extraction conditions, as well as the nature of the solvents, on two conventional flax gum extraction processes in order to reduce the amount of solvents used while maximizing yield using response surface methodology (RSM). In addition, it assesses the chemical and emulsifying properties of the gums obtained, giving an insight into their potential applications in various industries.

## 2. Materials and Methods

### 2.1. Plant Material

Mature flaxseeds (*L. usitatissimum* L.) were collected from the local market in the city of Kenitra located in the northwest of Morocco following the 2020-2021 harvest season. They were cleaned of all impurities (remains, stones, debris, roots, etc.), washed, and air-dried before being kept dry in clean, hermetically sealed glass bottles in a cool place until use.

### 2.2. Isolation and Extraction

In order to determine the most convenient procedure of extraction, two modified conventional methods based on hot water extraction were chosen. In brief, these methods include a seed pretreatment step by soaking in water or in a water/solvent mixture which was intended to soften the husk and help the extraction process of the oily part of the husk. This was followed by a drying step and then a sample preparation step to remove seed coat mucilage before the final separation and fractionation step to isolate the pure gum.

#### 2.2.1. Pretreatment

The goal here is to prepare the samples for the future flaxseed gum extraction stages, rather than to separate the oil from the husk completely. A pretreatment step was carried out inspired by Liang et al.'s 2020 work [24], using a water/benzoic acid mixture instead of ethanol because their ethanol-based dehulling procedure resulted in significant losses and caused the formation of two layers of gum in the subsequent extraction steps: an upper layer that is typically easier to extract and a lower layer containing oily impurities, which posed extraction challenges. To illustrate the pretreatment impact, two separate treatments were applied to different groups of seeds. One portion of the seeds (designated as pretreatment = “Yes”) was soaked in a 3 : 1 (v/v) solution of water and benzoic acid (1N) at room temperature for 12 hours. The oily components were subsequently removed by washing the seeds three times with a 1 : 3 (v/v) water/acetone solution. In contrast, the remaining portion (designated as pretreatment = “No”) had the seeds steeped in water alone for 12 hours at room temperature before being cleaned with sterile water. Subsequently, all seeds were dried and stored until further use.

#### 2.2.2. Seed Preparation

The seeds were either thoroughly crushed slightly with a hammer (intact), ground to a powder (blender), or whipped (glass beads). For the whipped procedure, the whole seeds were placed in a plastic container holding water at a 10 : 1 (v/w) ratio, along with a few glass beads, and briskly shaken before being let to stand for around 1 min. The operation is done three times (with a 1 min cycle) until a gelatinous mass appears. It should be noted that no purification step was performed on the powders acquired from the seed preparation step, and this will be the case throughout the remainder of this study.

#### 2.2.3. Solvents and Extraction Methods

Two different procedures of separation on and splitting were chosen to isolate the gum with some modifications while respecting the operating parameters such as time, temperature, and pH given by the authors.

#### 2.2.4. Method A

Method A is based on the procedure described by Kaushik et al., with some modifications [25]. The dried crushed/powdered or whipped seed sample of *L. usitatissimum* (50 g) was boiled for 2 h at 80°C with continuous stirring into 1 L of deionized water or mixed with a solution of ethyl alcohol/water 1 : 1 (v/v), and the pH was adjusted to 7. The extract was filtered through mousseline cloth and concentrated to approximatively 30% of the volume using rotary evaporator at 40°C, and then the concentrated extract obtained was precipitated with 1 : 1 volume of ethanol 95% solution (v/v). The precipitate was collected by centrifugation at 6000 rpm for 5 min followed by lyophilization.

#### 2.2.5. Method B

Method B that was used to extract gum from flaxseed hulls was described by Qian et al., [26]. The sample (50 g) was dispersed into deionized water (700 mL) or ethyl alcohol/water at 70°C for 3 h with stirring. The recovered supernatant was then centrifuged at 4000 rpm for 15 minutes to remove insoluble residues. These residues were washed twice by 5 : 1 volume of deionized water, and the extraction process was repeated as before until the pomace was exhausted (2 to 3 times). The supernatants were recovered using a vacuum pump, combined, were carefully poured into an acetone solution until no obvious gel precipitate appeared, and then left to settle for 12 h at room temperature. Then the obtained precipitate was centrifuged and washed with deionized water three times. After adding a 1:1 amount of ethanol 95% solution (v/v) to the mixture and repeating three washings, the crude precipitate was produced. The resulting sample was then left to dry in Petri dishes at room temperature before being freeze-dried for later use.

### 2.3. Experimental Design and Statistical Analysis

In order to investigate the impact of the selected variables on flax gum yield, it was decided for the rest of the study to work under high-temperature conditions and with the normal time that each method can take as mentioned in several works [27, 28].

#### 2.3.1. Full Factorial Design

In order to investigate the impact of the pretreatment, the preliminary screening related to the preparation of the seed before use, and prepared the solvent used in the extraction procedures chosen. For this purpose, a completely randomized 3 × 2 × 2 × 2 factorial experimental design was applied in the first stage to exhibit the influence of the independent qualitative variables at different levels, while the yield (%) was considered as an experimental response for a total of 25 combinations [21]. The selected factors are represented in Table 1.

#### 2.3.2. Response Surface Methodology (RSM)

In the second stage of the study, a response surface methodology (RSM) was used twice to find the effect of continuous variables on the yield (%) and to predict a formula with highest responses as target. A Box–Behnken design with three center points was employed twice due to lack of fit on the first attempt. There were two levels for each independent factor: pH (4–10), water to pretreatment solvent ratio (3:1 to 5:1), and extraction solvent ratio (1:1 to 3:1) [29]. The experimental design included star points and 3 central points for a total of 15 combinations chosen randomly. The response function was extraction *y* = yield (%). The related RSM design for the continuous independent variables is represented in Table 2.

#### 2.3.3. Statistical Analysis

Experiments were performed in triplicate, and the multiple linear regression was evaluated via the one-way analysis of covariance (ANCOVA) which blends the principles of regression analysis of variance (ANOVA) and regression. Single and interactive effects were determined to explore the significance of differences at *p* < 0.05. Using Hsu's MCB (Multiple Comparisons with the Best) method, the aim of maximising extraction yield was set, and the mean values of each group were compared to the highest mean value. The fitting model adequacy was checked by the coefficient of determination *R*^2^ (R-square) defined as the ratio of the explained variation to the total variation according to its magnitude. All statistical data analysis, regression model, and generated experimental designs were assisted by JMP® Pro 16.2.0 software program.

### 2.4. Characterization of Flaxseed Gum

#### 2.4.1. Determination of Extraction Yield, Moisture, Ash, and Protein Contents

The yield of *L. usitatissimum* gum *Y* (%) was calculated as the dry weight of the gum relative to the whole seed weight as below:

(1) $$\begin{array}{c} Y\;\left(\%\right)=\frac{M_{g}}{M_{s}}\times 100, \end{array}$$

where *M*_*g*_ is the mass (g) of extracted gum and *M*_*s*_ is the mass (g) of *L. usitatissimum* seed. Subsequently, the moisture content was determined from weight loss after heating 0.5 g of the extracted gum for 24 h at 105°C in an oven and the ash content was quantified after dry mineralization (3 g) at 550°C for 5 h in a muffle furnace according to the AOAC method [30].

The total protein content was estimated using the Bradford method (1976) [31] by adding 0.5 ml of Bradford reagent (BRADFORD REAGENT-B6916 from Sigma-Aldrich) to 0.5 ml of sample. After a 30 min dark rest at room temperature, absorbance was measured at 595 nm (Spectrophotometer UV-Visible CPS-240A, Shimadzu) against a bovine serum albumin (BSA) standard range of 0 to 0.1 g/L.

The total carbohydrate content of the polysaccharide hydrolyzate was determined according to phenol sulphuric acid method of Dubois et al. [32] by shaking 0.1 g of the samples with 1 mL of concentered H_2_SO_4_ for 30 min in a water bath at 45°C. Then, 5% of phenol was added and the mixture was heated for 5 min at 90°C. After cooling at room temperature, the absorbance was measured at 490 nm. A series of glucose concentration were used as standard.

#### 2.4.2. Fourier-Transform Infrared Spectra (FT-IR) Analysis

The principal functional groups were detected by analysis of infrared spectra using a Bruker Tensor II FT-IR Spectrometer system (Bruker Optics GmbH). About 5 mg of purified polysaccharide sample was mixed with 100 mg of anhydrous potassium bromide and pressed into a 13 mm disc. The IR spectrum was recorded in the range 400 to 4,000 cm^−1^ with a resolution of 4 cm^−1^.

#### 2.4.3. Emulsifying Properties

Oil-in-water emulsions were prepared by adding 5 ml of sunflower oil to 45 ml of hydrocolloid suspensions (60 mL) with a 0.5% (w/v) concentration previously prepared by dispersing the freeze-dried samples 2 h into distilled water with continuous stirring at room temperature until complete dissolution. The mixture was stirred at 1200 rpm for 5 min and homogenized at 9800 rpm for 2 min using high shear homogenizer system (CAT M. Zipperer GmbH Drive motor Unidrive X 1000D, Germany) and then sonified for 5 min by using an ultrasonic bath (model JP Selecta Ultrasons-HD, 3000867) [33]. The dispersions were then finally centrifuged at 4000 rpm for 10 min. The emulsifying capacity (EC) was calculated as

(2) $$\begin{array}{c} \text{EC }\left(\%\right)=\left(\frac{e_{v}}{t_{v}}\right)\times 100, \end{array}$$

where *e*_*v*_ is the emulsion volume and *t*_*v*_ is the total volume.

The emulsion stability (ES) against high temperatures was determined by heating in a water bath at 80°C for 30 min, followed by centrifugation at 3500 rpm for 5 min. The emulsion stability was calculated as [34]

(3) $$\begin{array}{c} ES\;\left(\%\right)=\left(\frac{f_{\text{ev}}}{i_{\text{ev}}}\right)\times 100, \end{array}$$

where *f*_ev_ is the final emulsion volume and *i*_*ev*_ is the initial emulsion volume.

## 3. Results and Discussion

### 3.1. Full Factorial Design

#### 3.1.1. Statistical Analysis

**Figure 1:**
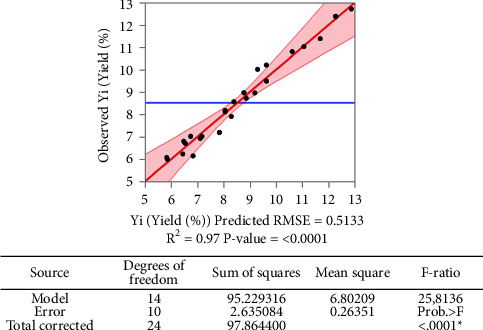
Scatterplot of fitting model for the full factorial design.

The high coefficient of determination *R*^2^ of 0.97 indicates that 97% of the variance in the yield can be explained by the model (Figure 1). The analysis of variance for the least-squares fit of the extraction yield variation model (%) shows an F-ratio of 25.8136 which indicates significant variation in the model relative to the error, while the probability associated with the *F*-ratio (Prob. > F) is very close to zero (“<0.0001”), meaning that the model has a significant effect relative to the error with no lack of fit.

#### 3.1.2. Effects of the Categorical Variables on the Extraction Yield

**Figure 2:**
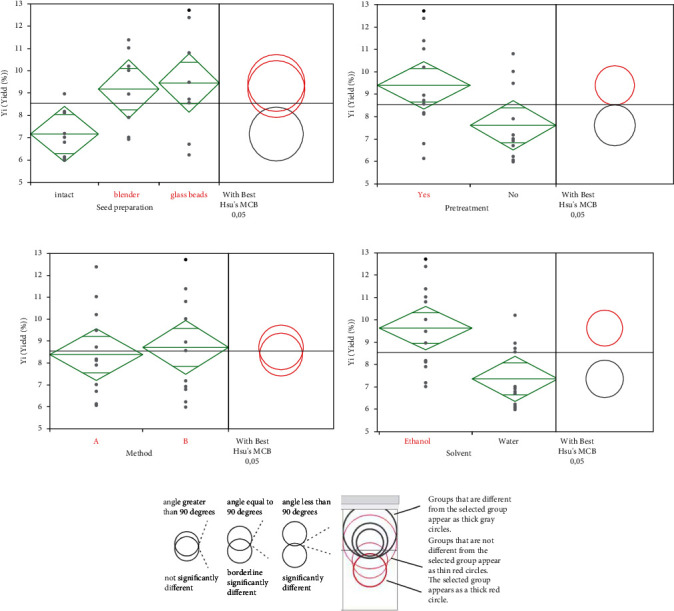
ANOVA analysis with Hsu's MCB test for each group. (a) One-way ANOVA of Yi (yield (%)) versus seed preparation. (b) One-way ANOVA of Yi (yield (%)) versus pretreatment. (c) One-way ANOVA of Yi (yield (%)) versus extraction solvent. (d) One-way ANOVA of Yi (yield (%)) versus extraction method. (e) Angles of intersection and significance with the graphical comparison circles.

The significant different factors and interactions affecting the extraction yield (%) were investigated, and the summary is given in Figure 2; notably, “Seed preparation,” “Pretreatment,” and “Solvent” emerged as key factors with highly significant effects, all boasting *p* values well below the conventional threshold of 0.05. This signifies their substantial influence on the dependent variable, underscoring the critical importance of seed preparation methods, pretreatment procedures, and solvent choices in our study. Furthermore, two interactions, “Seed preparation *∗* Pretreatment” and “Seed preparation *∗* Solvent,” were also deemed statistically significant, suggesting that the impact of seed preparation is contingent on both pretreatment and solvent selection. Also, the Method *∗* Solvent can be considered a small-scale effect interaction. However, the “Method” factor and the remaining interactions did not exhibit significant effects on the dependent variable, as indicated by their higher *p* values. These results contribute to a more nuanced understanding of the variables at play in our study, aiding in the refinement and fine-tuning of future experimental procedures.

Figures 2(a)–2(e) show the comparisons between various factor levels using ANOVA and Hsu's MCB test, highlighting the maximum and minimum p values for each comparison.

The thickness of the gray circles is proportional to the mean difference from the selected group (red circles), and the outside angle of intersection indicates whether the group means are significantly different as shown in Figure 2(e).

The analysis of variance (ANOVA) for the “Seed preparation” factor, which has three levels, revealed significant results regarding its influence on the dependent variable. The data show a statistically significant difference among the sample preparation levels (“Intact,” “Blender,” and “Glass Beads”). The calculated F-ratio is 4.2041 with a *p* value of 0.0284, indicating that the choice of sample preparation method has a substantial impact on the studied variable.

Furthermore, comparisons suggest that the “glass beads” and “blender” levels, with *p* values of 0.7738 and 0.5380, respectively, do not have a significant effect compared to the best level (“Intact”). However, the “intact” level shows a significant effect compared to the worst level in terms of the studied variable. This means that using “Glass Beads” or “Blender” for sample preparation has a different and significant effect compared to “Intact.” Nevertheless, the choice between “Glass Beads” and “Blender” appears to have no significant impact on one another, but both significantly differ from “Intact.”

Similarly, the “Pretreatment” factor (*p* value = 0.0238) and the “Solvent” factor (*p* value = 0.0027) have effects less than 0.05, signifying significance on the dependent variable. The comparison between “Yes” and “No” levels for the “Pretreatment” factor demonstrates a statistically significant difference, with a difference greater than −3.2974 at a 95% confidence level. Likewise, the comparison between “Ethanol” and “Water” levels of the “Extraction Solvent” factor shows a statistically significant difference greater than −3.6701 at a 95% confidence level. However, neither the analysis of variance (ANOVA) nor the direct comparison between “A” and “B” levels of the “Method” factor indicates a significant difference at the 95% confidence level. Consequently, the “Method” of separation does not have a significant effect on the dependent variable.

Following these results and due to availability of material and efficiency, for the subsequent stages of the study, For the extraction of FG, we chose technique “A,” which involves “pretreatment,” “glass bead sample preparation,” and “ethanol” usage as solvent. This approach will yield high-quality results by optimizing the extraction conditions.

### 3.2. RSM Results

In order to optimize the FG extraction process and identify the optimal circumstances for maximal output, our research was divided into two crucial stages. We carefully selected variables in the first phase that had the greatest potential to enhance the extraction procedure. The second phase involved precisely determining the optimal extraction conditions while accounting for the following variables: the medium pH of extraction technique A, the water/benzoic acid ratio of the pretreatment step, and the ethyl alcohol/water ratio. It is important to remember that different combinations of these factors could produce the best FG production.

#### 3.2.1. Statistical Analysis for the Model Fitting

**Figure 3:**
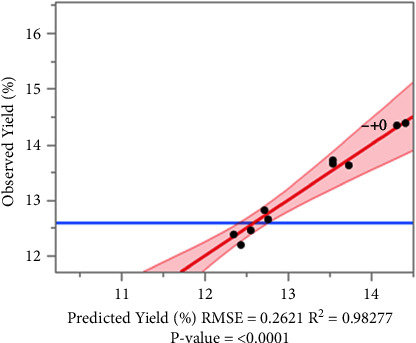
Scatterplot of observed values versus predicted values for RSM.

The response surface analysis indicates that the generated response surface curves accurately predict the extraction yield as a function of variations in the independent variables: water/benzoic acid ratio, ethyl alcohol/water ratio, and pH. The results reveal a high-quality model with a high coefficient of determination *R*^2^ of 0.98277, indicating that our model explains nearly 98% of the observed variation in yield. The low Root Mean Square Error (RMSE) of 0.2621 strengthens the precision of our predictions. Furthermore, the extremely low *p* value of <0.0001 underscores the statistical robustness of our model, suggesting that the relationship between our independent variables and extraction yield is highly significant (Figure 3).

#### 3.2.2. Effect of Different Variables on Yield

Sequential testing following the analysis of variance (ANOVA) results shows that the factors water/benzoic acid ratio, ethyl alcohol/water ratio, pH, and some of their interactions have significant effects on the response (Yield %) (Table 3). The water/benzoic acid ratio factor exhibits an F-ratio of 31.5662 with a probability (Prob. > F) of 0.0014, indicating a significant effect on the response (Yield %). Similarly, the ethyl alcohol/water ratio factor has an F-ratio of 20.1605 with a probability of 0.0041, signifying that its variation has a statistically significant impact on yield. However, the very high F-ratio of 181.2324 with an extremely low probability, less than 0.0001, indicates that the pH factor has an extremely significant effect on the response. Additionally, interactions between the water/benzoic acid ratio and ethyl alcohol/water ratio, water/benzoic acid ratio and pH, ethyl alcohol/water ratio and pH, and water/benzoic acid ratio *∗* ethyl alcohol/water ratio have significant probabilities, revealing how these factors interact to influence the response.

**Figure 4:**
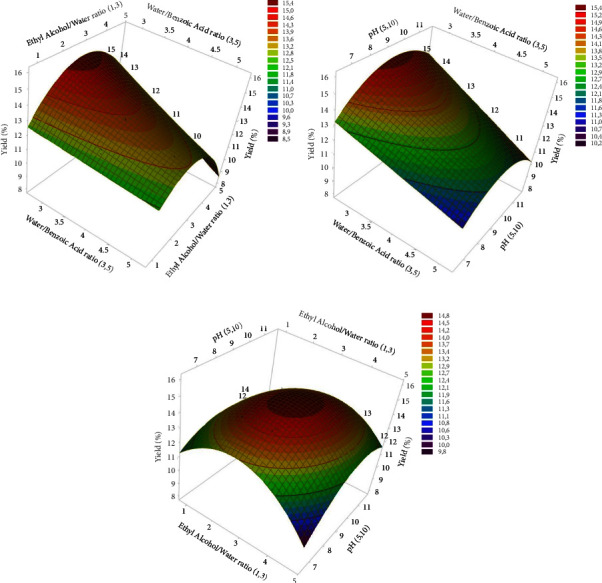
Graphical representation of 3D response surface plots illustrating the interactive effects of key variables on yield (%) of flaxseed gum. (a) Surface plot for variation in water/benzoic acid ratio and ethyl alcohol/water ratio. (b) Surface plot for variation in water/benzoic acid ratio and pH. (c) Surface plot for the variation in ethyl alcohol/water ratio and pH.

The three 3D response surface curves (Figure 4) illustrate the impact of the water/benzoic acid ratio, ethyl alcohol/water ratio, and pH factors, highlighting the complex interactions between the factors and their influence on yield. In general, the observed curvilinear effect on these curves results from quadratic interactions. The first curve reveals a concave relationship between water/benzoic acid ratio and ethyl alcohol/water ratio concerning yield, emphasizing a significant effect on yield, as confirmed by sequential ANOVA testing. The yield increases as the water/benzoic acid ratio increases, reaching a peak at around 3.262. However, this curve also indicates the importance of quadratic interactions, ethyl alcohol/water ratio *∗* ethyl alcohol/water ratio, with a probability of 0.0101. Likewise, the observed curvilinear effect on the second curve is due to the significance of the pH *∗* pH quadratic effect on the response, with a very low probability (<0.0001) and an optimal point at around 3.001 for ethyl alcohol/water ratio, suggesting that yield is optimal at this value. However, this relationship is modulated by pH, with the maximum yield achieved at a pH of approximately 8.731. Furthermore, the third curve confirms the significant impact of pH on yield, with its concave shape and a peak, suggesting that yield depends on these two factors, and there exists an optimal combination of ethyl alcohol/water ratio and pH values that maximizes yield.

### 3.3. Optimization

The model we developed to predict the relationships between independent variables and the dependent variable, yield, is based on a complete quadratic equation. This equation accounts for interactions and quadratic effects of factors, providing precise modeling of the expected response. The prediction equation for yield is as follows:

(4) $$\begin{array}{c} \text{Yield}=1.2474\times X_{1}-1.2221\times \left(X_{1}^{2}\right)-0.5206\times X_{2}+0.4160\times X_{3}-0.5252\times \left(X_{3}^{2}\right)-0.3260\times \left(X_{1}\times X_{2}\right)-0.3566\times \left(X_{2}\times X_{3}\right), \end{array}$$

where

1. *X*_1_ (pH): the level of pH.
2. (*X*_1_^2^): the square of the level of pH.
3. *X*_2_ (water/benzoic acid ratio): the ratio of water to benzoic acid.
4. *X*_3_ (ethyl alcohol/water ratio): the ratio of ethyl alcohol to water.
5. (*X*_3_^2^): the square of the ethyl alcohol/water ratio.
6. *X*_1_ × *X*_2_: the interaction between pH and the water/benzoic acid ratio.
7. *X*_2_ × *X*_3_: the interaction between the water/benzoic acid ratio and the ethyl alcohol/water ratio.

The positives coefficients *X*_1_ (1.2474) and *X*_3_ (0.5252) suggest that increased pH levels and the ethyl alcohol/water ratio generally result in larger yields, whereas *X*_2_ exhibits a negative influence (−0.5206), implying that higher water to benzoic acid ratios lead to lower yields. Furthermore, it appears from the negative coefficients −0.3260 and −0.3566 for the interaction terms *X*_1_ × *X*_2_ and *X*_2_ ×  *X*_3_ that certain combinations of pH levels and water-to-benzoic acid ratios, as well as some combinations of water/benzoic acid and the ethyl alcohol/water ratios lead to lower yields.Underscoring the significance of optimizing these variables to enhance the extraction process and improve overall yield.

Utilizing this response surface model, we plan to achieve a maximize yield of 14.51% and minimize resource consumption by conducting experiments and analyzing key variables such as water/benzoic acid ratio at 3.2 to 1, ethyl alcohol/water ratio at 2.17 to 1, and pH at 8.7.  Table 4 summarizes the predicted and experimentally validated values for the factors.

Results derived from the prediction model were experimentally validated, and the predicted values closely matched the experimental measurements. For instance, the predicted yield was computed as 14.51087%, while experimental measurements yielded 14.47% with a margin of error of ±0.14 after five replicates. The strong match between predicted values and experimental outcomes demonstrates our model's robustness and reliability.

By changing the extraction process conditions as provided by the optimization model, we can potentially achieve a yield that exceeds 14%, which is higher than that found by Hu et al. (12.73%) at higher temperatures (70°C–90°C), using more resources and requiring much longer periods of time [27]. The precision of predictions, coupled with the minimal difference between expected values and experimental measurements, suggests that our model can be a valuable tool for the FG extraction industry, enabling the optimization of production conditions to achieve high and consistent yields.

### 3.4. Characterization of Flaxseed Gum

#### 3.4.1. Determination of Extraction Yield, Moisture, Ash, and Protein Contents

The chemical compositions of flaxseed gums obtained by the optimization process (OFS) in comparison with a fraction that underwent a purification step after washing and precipitation three times with isopropyl alcohol (PFS) are presented in Table 5. The yield of the OFS was 14.47% of the seed weight, while the yield of the PFS was 12.44 equivalent to 85.97% of the unpurified gum. Total sugars, moisture, and ash levels increased drastically after purification, while total nitrogen content decreased slightly from 51.02 mg/g to 50.74 mg/g of gum.

### 3.5. FT-IR Spectra Analysis

The results of FT-IR spectroscopy reveal characteristic bands in the fingerprint region of gum, as reported in previous studies [3, 35]. A broad intense band at 3392 cm^−1^ is attributed to the stretching vibrations of hydroxyl groups (−OH), indicating the presence of hydrogen bonding in the structure [36]. Another noteworthy band is the asymmetric stretching vibration at 2923 cm^−1^, which is due to C-H stretching. The band at 1716 cm^−1^ is consistent with the vibration of a nonconjugated carbonyl group (C=O). Furthermore, the bands observed between 1630 cm^−1^ and 1400 cm^−1^ suggest the absorption of carbonyl groups (C=O) originating from the carboxyl function of galacturonic acid, with an elongation vibration observed at 1373 cm^−1^, as well as carboxylates (-COO-) corresponding to uronic acids [37]. Bands located between 1151 cm^−1^ and 962 cm^−1^ indicate the presence of C-O and C-O-H bonds, suggesting the presence of glycosidic xyloglucan [38]. Additionally, the bands at 742 cm^−1^ and 675 cm^−1^ can be attributed to the anomeric configuration linkage units in the pure crystallinity of gum structure [39].

**Figure 5:**
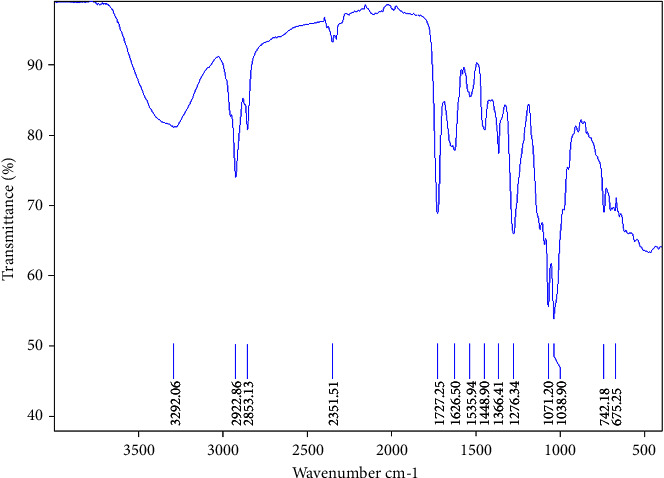
Flaxseed gum FT-IR spectra.

However, our findings were slightly different from those reported by Ren et al. Despite showing typical polysaccharide absorption peak areas [18], specific vibrations associated with the C-H bond were observed in the frequency range of 2900 cm^−1^ to 2800 cm^−1^ (Figure 5). While this may suggest the presence of benzyl groups (C_6_H_5_-CH_2_-), the presence of vibrations of the C=C bond around 1600 cm^−1^, along with the absence of the characteristic N-H bending of the amide II band of proteins, precisely the absence of the double high-frequency stretching C=O bands and low-frequency N-H bending, favors the possibility of traces of benzoic acid resulting from the pretreatment step. This is in line with the low protein content previously found in the chemical composition of the OFS and PFS, demonstrating the success of the deproteinization process. Further purification may be required to confirm this hypothesis.

### 3.6. Emulsifying Properties

**Figure 6:**
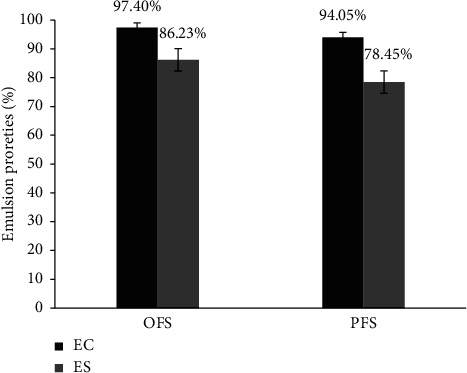
Emulsification properties of flax gum solutions.

The emulsification properties of flax gum solutions were evaluated before and after purification treatment. As shown in Figure 6, both OFS and PFS had excellent emulsifying capacities (EC) well in excess of 94%, with mean values of 97.40 ± 0.49% and 94.05 ± 0.10%, respectively. This can be explained by the low partial denaturation of lipoproteins in the hydrocolloid chains, which prevents the formation of nanogels at pH 8 and consequently greater aggregation of emulsion droplets at the oil/water interface [40]. Additionally, the pretreatment procedure may play a significant role in the release of particular bioactive components that effectively improve FG's apparent viscosity. This improvement is important for FG's rheological and functional properties, which direct its production and practical uses as a stabilizer, thickener, gelling agent, texture modifier, and suspending agent [24].

However, emulsification stability (ES) decreased drastically, particularly after isopropyl alcohol treatment, with value of 86.23 ± 0.68%, while 78.45 ± 0.35% for PFS (optimization process). A study of the steric and mechanical behavior of high molecular weight hydrophilic polysaccharide chains in galactomannans reported the formation of layers around oil droplets, preventing aggregation of the emulsifier [41]. The slight decrease in emulsification stability suggests that the enhanced level of polymerization products inhibits the mobility of the molecules and consequently slows down absorption at the oil/water interface [27].

## 4. Conclusions

This study uses two hot water procedures to illustrate the critical necessity of extraction conditions for flaxseed gum yield. The results of the factorial design demonstrated that pretreatment, seed preparation, and solvent choice play a vital role in extraction yield. Furthermore, the use of a response surface methodology (RSM) focusing on continuous variables allowed us to determine the optimal parameters for maximizing gum yield, achieving a key value of 14.47%. Additionally, this study conducted an in-depth analysis of the chemical and emulsifying properties of the gum, offering promising prospects for its use as an ingredient in various products, especially in the food industry. However, it is worth noting that the optimization of other parameters such as time and additional purification steps remains a key factor to meet the specific requirements of certain industrial-scale applications.

## Figures and Tables

**Table 1:** The independent factors and their levels for the full factorial design.

Factors	Levels	Values		
		Lv1	Lv2	Lv3
Pretreatment	2	Yes	No	
Seed preparation	3	Intact	Blender	Glass beads
Solvent	2	Ethanol/water	Water	
Method	2	A	B	

**Table 2:** Box–Behnken central composite design for the continuous variables.

Run	Factors		
	Water/benzoic acid ratio	Ethyl alcohol/water ratio	pH
1	4 : 1	3 : 1	10
2	5 : 1	2 : 2	10
3	4 : 1	1 : 1	10
4	3 : 1	2 : 1	10
5	5 : 1	3 : 1	7.5
6	4 : 1	2 : 1	7.5
7	4 : 1	2 : 1	7.5
8	4 : 1	2 : 1	7.5
9	3 : 1	1 : 1	7.5
10	5 : 1	1 : 1	7.5
11	3 : 1	3 : 1	7.5
12	5 : 1	2 : 1	5
13	4 : 1	1 : 1	5
14	4 : 1	3 : 1	5
15	3 : 1	2 : 1	5

**Table 3:** Analysis of variance (ANOVA) for the predictive model of extraction yield (%).

Source	Sequential sum of squares	*F*-ratio	Prob. > *F*
Water/benzoic acid ratio (3, 5)	2.168144	31.5662	0.0014^*∗*^
Ethyl alcohol/water ratio (1, 3)	1.384732	20.1605	0.0041^*∗*^
pH (5, 10)	12.448042	181.2324	<0.0001^*∗*^
Water/benzoic acid ratio *∗* ethyl alcohol/water ratio	0.508535	7.4038	0.0346^*∗*^
Water/benzoic acid ratio *∗* pH	0.425033	6.1881	0.0473^*∗*^
Ethyl alcohol/water ratio *∗* pH	0.304326	4.4307	0.0799
Ethyl alcohol/water ratio *∗* ethyl alcohol/water ratio	0.715965	10.4238	0.0179^*∗*^
pH *∗* pH	5.547263	80.7632	0.0001^*∗*^

**Table 4:** Predicted and experimentally validated values for the factors.

Factors	Predicted model	Experimental validation
Water/benzoic acid ratio	3.189 : 1	3.2 : 1 ± 0.05
Ethyl alcohol/water ratio	2.137 : 1	2.17:1 ± 0.05
Medium pH	8.7	8.7 ± 0.03
Yield (%)	14.51087	14.47 ± 0.04

**Table 5:** Chemical comparison of two flax gum solutions.

Gums	Component				
	Yield (%)	Moisture content (%)	Ash content (%)	Total protein content (mg/g gum)	Total carbohydrate content (mg/g gum)
OFS	14.47 ± 0.04	7.31 ± 0.03	11.07 ± 0.05	51.02 ± 0.41	578.83 ± 2.33
PFS	12.44 ± 0.18	9.24 ± 0.02	12.75 ± 0.15	50.74 ± 0.25	619.09 ± 2.90

## Data Availability

The data used to support the findings of this study are included within the article.
